# Does cognitive aging follow an orchid and dandelion phenomenon?

**DOI:** 10.3389/fnagi.2022.986262

**Published:** 2022-10-10

**Authors:** Emma A. Rodrigues, Gregory J. Christie, Faranak Farzan, Sylvain Moreno

**Affiliations:** ^1^School of Interactive Arts and Technology, Simon Fraser University, Surrey, BC, Canada; ^2^Circle Innovation, Surrey, BC, Canada; ^3^School of Mechatronics and Systems Engineering, Simon Fraser University, Surrey, BC, Canada

**Keywords:** healthy aging, cognitive trajectories, lifestyle factors, environmental susceptibility, cognitive aging

## Abstract

Cognitive reserve reflects the brain’s intrinsic adaptive capacity against the neurodegenerative effects of aging. The maintenance or enhancement of the brain’s cognitive reserve plays a crucial role in mitigating the severity of pathologies associated with aging. A new movement, social prescribing, which focuses on prescribing lifestyle activities as a treatment for patients, is growing in popularity as a solution against aging pathologies. However, few studies have demonstrated a clear impact of lifestyle activities on individual cognitive health, outside of floor and ceiling effects. Understanding *who* benefits from *which* lifestyle factors remains unclear. Here, we investigated the potential effects of lifestyle activities on individuals’ cognitive health from more than 3,530 older adults using a stratification method and advanced analysis technique. Our stratification methods allowed us to observe a new result: older adults who had relatively average cognitive scores were not impacted by lifestyle factors. By comparison, older adults with very high or very low cognitive scores were highly impacted by lifestyle factors. These findings expand the *orchid and dandelion* theory to the aging field, regarding the biological sensitivity of individuals to harmful and protective environmental effects. Our discoveries demonstrate the role of individual differences in the aging process and its importance for social prescribing programs.

## Introduction

The search for healthy longevity is likely to be the flagship project of the coming century (Harrar, 2019). The interest in the cure for aging is obvious. As we age, we become increasingly vulnerable to the effects of excessive metabolic loads usually associated with trauma, ischemia, or neurodegenerative processes (Toescu, 2005). How we cope with those changes is a central concern in our modern society (Christensen et al., 2009). Thousands of clinical drug trials have been conducted to reverse or reduce aging symptoms, but thus far, drug treatments have shown little or no efficacy (Casey et al., 2010; Morales-Navarro et al., 2019). Therefore, one of the most effective prevention mechanisms against the neurodegenerative effects of aging lies in maintaining or enhancing the brain’s cognitive-reserve capacity (Stern, 2002, 2003). Cognitive reserve capacity reflects the difference between age-related brain deterioration and observed clinical outcomes (Cabeza et al., 2018). It’s decline has been associated with behavioral severity of aging symptoms that result from neurodegenerative processes (Tucker and Stern, 2011).

Lifestyle factors have been shown to benefit cognitive reserve, but their impact seemed to vary across individuals (Krivanek et al., 2021; Soldan et al., 2021). The “*one size fits all*” approach regarding strategies to promote brain health currently dominates the literature. However, many studies have resulted in confusing results and small-sized effects (Christie et al., 2017). This scientific confusion leads to unclear strategies on how good cognitive health can be promoted. Some factors have been more consistently studied and have produced more robust evidence regarding their potential benefits such as some forms of cognitive, social, and physical activities. However, most studies focus on only one or two lifestyle factors. Given the individual and multifactorial nature of cognitive health, understanding *who* benefits from *which* lifestyle factors remains one of the central questions of aging research.

For several decades, researchers have explored the potential impact of many lifestyle factors such as education (Anstey and Christensen, 2000; Cagney and Lauderdale, 2002), exercise (Anstey and Christensen, 2000; Hillman et al., 2008), or meditation (Lazar et al., 2006; Moore and Malinowski, 2009) as promoters of cognitive reserve capacity. Results have shown reductions in the incidence of aging symptoms, advocating for this avenue of research as having a greater potential for impact on the quality of life than disease-specific approaches (Kaeberlein et al., 2015; Fratiglioni et al., 2020). Evidence in the literature also suggests that if environmental and/or modifiable factors are causally linked to changes in cognitive function, the cognitive trajectory should be affected by changes in the occurrence of these factors (Kivipelto et al., 2018). Despite these benefits being inconsistently observed, the contribution of modifiable lifestyle factors to cognitive health means that there may be potential to stabilize or improve declining trajectories of cognitive function (Clare et al., 2017). Understanding these relationships could have important benefits for cognitive health in later life and serve to mitigate the risks of higher genetic risk (Rovio et al., 2005).

Here, we investigated this question using stratification, a method that considers heterogeneous populations and subdivides them into more homogeneous subgroups. This method has been previously used in clinical and animal models, and has resulted in key findings for aging research (Zullo et al., 2019). The approach ensures a similar number of individuals and more homogenous samples in each of the cognitive categories reducing the likelihood of one category being more probable than the other. Further, this approach allows researchers to isolate and measure effects within one category, while not affecting the other categories. Here, we applied this method to a large, heterogeneous longitudinal dataset, the Health Retirement Study (HRS), enabling us to explore the impact of life factors on the aging trajectories of different groups of older adults (Health and Retirement Study, Public Use Dataset, 2018). Indeed, recent findings highlight that the shape and course of cognitive health in adulthood are best understood as a range of potential developmental trajectories that reflect person-specific characteristics and environmental opportunities and constraints (Hertzog et al., 2009). For our analysis, we chose a standardized and well-regarded clinical test called Word Recall (Morris et al., 1989) as a cognitive health proxy. This measure was selected given that memory function is one of the first cognitive capacities to decline with age. In specific, working memory has been recognized to play a critical role in many cognitive tasks and it’s decline can impact a variety of cognitive measures, making it a crucial function to consider (Salthouse and Meinz, 1995). Furthermore, this particular assessment is less susceptible to both ceiling and floor effects, making it a sensitive measure for analysis (see Supplementary material for additional justification).

The goal of this study was to investigate who benefits from which lifestyle factors. Using this framework, we focused on three main hypotheses: (i) *Enriching* lifestyle factors such as exercise, social activities and educational training would lead to enhanced cognitive scores (e.g., Berezuk et al., 2017; Guerra-Carrillo et al., 2017), and (ii) *depleting* lifestyle factors such as smoking, sedentarism and low financial stability would lead to lower levels of cognitive scores (e.g., Schumpf et al., 2017; Schroeder et al., 2019). Finally, the third and main hypothesis of this work is (iii) lifestyle factors would have different impacts on long-term cognitive health depending on participants’ *current* levels of cognitive health (e.g., a *depleting* factor will have more negative weight on an individual with a higher level of cognitive health than an individual with a lower level). To approach these questions, we used an ordinal logistic regression model followed by a marginal effects analysis to assess the relevance of these factors on individual differences in cognitive health. We anticipated that the stratification of the cognitive variable would provide richer information on the dynamics of cognitive decay by determining the relevant factors that positively or negatively impact cognition.

## Materials and methods

### Software and database

The software used was Stata 14.2.

The HRS is a longitudinal database that collects employment history, work history, disability, retirement plans, and net worth, income and health of U.S. population over the age of 50 (Health and Retirement Study, Public Use Dataset, 2018). Our goal was to use the HRS to evaluate variables preserving cognitive health in aging. We extracted cognitive health information along with life factors data from the HRS, as reported in the modules Preload, Physical Health, Leave-behind questionnaires, and Cognition of 2012 and 2016 waves. We selected the same individuals present in both waves, following a longitudinal approach. The dependent variable, cognitive health, was computed as the sum of the total number of words correctly remembered by the respondents during the immediate and delayed recalls in each of the waves. The creation of this composite measure allowed for a more fine-grained analysis of the memory function as the original variables’ scores ranged from 0 to 10, whereas the scores of the composite measure ranged from 0 to 20.

### Participants and selection criteria

As part of our selection criteria, we included individuals above the age of 60. In total, our representative dataset contained information on 20,500 respondents characterized by 36 independent variables. Given our research question and hypotheses, a substantive portion of the independent variables were focused on participants’ evaluations of their life circumstances, subjective wellbeing, and lifestyles. This information was obtained from a specific portion of the database referred to as the leave-behind questionnaire. This data is obtained in each biennial wave from a rotating 50% of the core participants who complete the in-person interview (Smith et al., 2017). Therefore, longitudinal data is available at 4-year intervals. As this data is not collected in person, and participants are asked to mail responses at a later time, the response rates decline significantly. We also excluded respondents who had missing data for any of these previously listed factors. Our final number of respondents was 3,530. Observations containing missing values were excluded. Details regarding this process and justification can be found in the Supplementary material. The data was strongly balanced with 1,752 male participants and 1,778 female participants.

### Variable selection

The selected lifestyle factors can be found in Table 1 and were identified based on existing literature (for references, see Supplementary Table 1). Supplementary Table 1 identifies key results in the literature that support the inclusion of these specific factors as relevant to cognitive health. Following this review of the literature, we decided to include 36 lifestyle factors.

**TABLE 1:** Description of 36 covariates included in analysis.

Covariates	
Ongoing health problems	Often do word games
Ongoing physical/emotional problem in spouse/child	Often play cards and games
Ongoing drug/alcohol problem with family member	Often do writing
Ongoing difficulties at work	Often use computer
Ongoing financial strain	Often do maintenance/gardening
Ongoing housing problem	Often bake/cook
Ongoing problems in close relationship	Often sew/knit
Often do activities with grandchildren	Often walk for 20 min
Often volunteer with youth	Often do vigorous activities
Often do charity work	Often do moderate activities
Often do education courses	Often do mild activities
Often attend non-religious organizations	Age
Often pray privately	Years of education
Often read	Smokes
Often watch television	Drinks
Often do hobby	Often attend sports/socials/clubs
Often care adult	Often play sports/exercise
Ongoing difficulty paying bills	Regularly help ailing family/friends

### Wave selection

The majority of the selected lifestyle variables observed in Table 1, belong to a section of data which is obtained by the HRS in the form of self-administered questionnaires that were left with respondents upon the completion of an in-person Core Interview. This portion of the data is referred to as the leave-behind questionnaire (LBQ). This set of data includes information about participants’ evaluations of their life circumstances, subjective wellbeing, and lifestyle (Smith et al., 2017). This data is obtained in each biennial wave from a rotating (random) 50% of the core panel participants who complete the in-person interview. Therefore, longitudinal data is available at 4-year intervals. At the time of the data cleaning and analysis, the two most recent waves were selected which were 2012 and 2016. Additional prior waves could have also been included but this would lead to a larger loss of observations given the longitudinal nature of our analytical approach. For more information on this section of data and for similar methodological approaches please see Han and Richardson (2015) and Smith et al. (2017), respectively.

### Stratification

The original ordinality of the dependent variable was reflected in the 10 categories representing the number of recalled words. Given the small number of observations in each of the original categories, we decided to merge them into groups of 20%. The bottom 20% was category 1, the second 20%, category 2, and so forth with the highest 20% being category 5. The sectioning process allowed us to define which data belonged to which category without the bias of the researcher. Furthermore, it avoided skewed results due to heterogeneous population concentrations.

### Model selection and description

To address the research question, we used an ordinal logistic regression (OLR) model. This model provides the most informative comparisons for the subject matter as well as the desired amount of model flexibility (Fagerland, 2014). In addition, due to the logistic nature of the distribution function, the exponential form of the regression coefficients can be interpreted as odds ratios (ORs) (Hosmer et al., 2013). The OLR is indicated when an originally continuous response variable is later grouped. This model allows us to compute the odds of an individual being at or below a category (Abreu et al., 2008). The framework of the ordinal regression model is described in the following section.

#### Ordinal logistic regression model

Given our ordinal response variable *y*, which represents the cognitive function, the categories can be ordered from a lower degree of cognition to a higher degree of cognition. *y*_*it*_ will take a discrete value between 1 and 5 corresponding to the five categories in our study. For the purpose of estimation of the ordered logit model, we consider the latent variable representation on Equation 1 (from the Supplementary material), where *X*_*it*_ is a *k*×1 vector of covariates, such as those described in Supplementary Table 1, and *u* is logistically distributed with Equation 2 (from the Supplementary material) (Greene and Hensher, 2010).


(1) yit*=βX′i⁢t+ui⁢t,i=1,…,N.

(2) $$\Lambda \,\left(z\right)=e^{z}/1+e^{z}$$

Regarding the thresholds for classification of the model outcome by the respective categories, we define Equation 3 (from the Supplementary material). Moreover, the conditional probability of an individual being in a given category is computed as Equation 4 (from the Supplementary material)


(3) $$Y_{i\,t}=\left\{ \begin{array}{c} 1\,i\,f\,y_{i\,t}^{*}\leq \alpha_{1}\\ 2\,i\,f\,\alpha_{1}<y_{i\,t}^{*}<\alpha_{2}\\ 3\,i\,f\,\alpha_{2}<y_{i\,t}^{*}<\alpha_{3}\\ 4\,i\,f\,\alpha_{3}<y_{i\,t}^{*}<\alpha_{4}\\ 5\,i\,f\,y_{i\,t}^{*}\geq \alpha_{4} \end{array}\right.$$

(4) Pr⁡[yi⁢t=1|X]=Λ⁢(α1-β⁢X′i)Pr⁡[yi⁢t=2|X]=Λ⁢(α2-β⁢X′i)-Λ⁢(α1-β⁢X′i)Pr⁡[yi⁢t=3|X]=Λ⁢(α3-β⁢X′i)-Λ⁢(α2-β⁢X′i)Pr⁡[yi⁢t=4|X]=Λ⁢(α4-β⁢X′i)-Λ⁢(α3-β⁢X′i)Pr⁡[yi⁢t=5|X]=1-Λ⁢(α4-β⁢X′i)

The OLR is the most commonly used logistic regression model for an ordinal response, given that the effect of a covariate on *y* can be quantified by one regression coefficient. Furthermore, it allows for the calculation of odds ratios in a clear and reliable manner which will assess the odds of an individual being in a given category instead of another. See, for instance, McCullagh (1980) for more details on the model estimation. Results on the OLR analysis can be found in the Supplementary Tables 2, 3 which present the overall estimation results.

## Results

Using an ordinal logistic regression (OLR) approach, we sought to determine the relevant factors that impacted the cognitive function of individuals. Our analysis included 36 prospective factors and a sample of 3,530 older adults. Observations containing missing values were excluded (see Supplementary material for further details). The factors that positively impact cognitive health and display stronger statistical significance (i.e., *p* <0.001) include several factors that appear to require greater levels of concentration, such as *Often read*, *Often do word games, Often sew/knit*, and *Often use computer;* physical activities such as *Often do mild activities;* as well as other factors such as *Years of Education* and *Drinking*. Other factors that also have a positive impact, although of smaller statistical significance, include *Often does hobby* (*p* = 0.043). Conversely, the factors *Often walk 20 min*, *Age*, *Ongoing housing problems*, and *Smoking* exhibit a negative impact on cognitive health, also with a strong statistical significance (i.e., *p* < 0.001). For the factors that did not display any statistically significant impact on the measure of cognitive health, please see the Supplementary material. Additional corroborating references are included for each covariate are also included in the Supplementary material.

**FIGURE 1:**
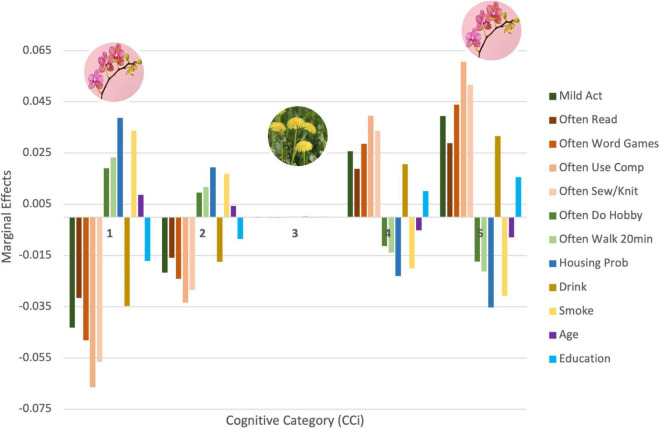
Relationship between lifestyle factors and different cognitive categories. The figure is color coded based on similarity of covariate. In dark blue are the factors related to financial strain, in shades of orange are the factors that require greater levels of concentration, in shades of green are the factors that relate to physical exercise, in purple and light blue is age and level of education, and in shades of yellow is smoking and drinking.

From the marginal effect analysis of our OLR model, we next sought to determine if the impact of these covariates (i.e., selected lifestyle factors) differed based on individual differences in cognitive health (hypothesis 3). We sought to discover the actual contribution of each factor to the likelihood of an individual remaining or migrating from a certain cognitive level. We therefore stratified participants into five non-overlapping cognitive categories (CC), based on their performance in the word recall test, with CC1 corresponding to the lowest level of cognitive function and CC5 the highest. We then computed the marginal effects of each covariate at each CC, the results of which are depicted in Figure 1. The figure presents the contribution of the significant covariates in each of the cognitive categories considered. The sum of the marginal effects of the same covariate across all categories is zero. Each significant covariate contributed positively (if greater than zero) or negatively (if smaller than zero) to an individual’s permanence in each category. For example, *housing problems*, represented in dark blue in Figure 1, has a positive relation with CC1. This indicates that having housing problems contributes positively to an individual remaining in this cognitive category. On the other end of the spectrum, the negative relationship with CC5 indicates that having housing problems contributes negatively to an individual remaining in this cognitive category. This analysis illustrates that the impact of lifestyle factors changes depending on the cognitive score of the individual. That is, the impact of lifestyle factors on long-term cognitive function depends on the current cognitive score of the individual. Similar interpretations can be made from the covariates *Walk 20 min*, *Age*, and *Smoking*. The opposite effect can be found with the following factors: *Reading*, *Word Games*, *Using Computer*, *Sewing/Knitting*, *Mild Activities*, *Years of Education*, *Hobby*, and *Drinking*. These factors contributed negatively to permanence in lower-level categories and positively in higher-level categories.

Figure 1 also illustrates how significantly lifestyle factors impact the polarized CCs, given the magnitude of the marginal effects. In this figure, we observe how the extreme values of the cognitive scale seem to be the most sensitive to the factors considered. This impact decreases as we move towards the central cognitive score. Finally, our results also show that the impact of lifestyle factors for people in CC3 is the smallest compared to the impact on other levels. To test the robustness of this result, the analysis was replicated with an equal number of participants per category and with different aggregates of cognitive scores, dividing the cognitive proxy into groups of 3, 4, and 9 cognitive levels. The pattern of impacts remained constant throughout all verifications (see Supplementary material for further details^1^).

## Discussion

Understanding how modifiable lifestyle factors may maintain or promote cognitive health can lead to a healthier aging population (Clare et al., 2017). Here, we report the impact of various lifestyle factors on individuals in five different, stratified cognitive categories (CCs). Consistent with previous findings, we found that increased aging is associated with declines in memory function, as increasing age is negatively associated with higher categories of memory function. Similarly, results associated with education are also in accordance with existing literature as increased degrees of education are associated with higher categories of cognition. Furthermore, previous results have also been identified in the presented results in which we observed that some of the remaining factors have detrimental (e.g., financial constraints or smoking) or enriching (e.g., reading daily or several times a week) effects on long-term memory function. There are two critical results to highlight from this study. First, the impact of these factors was not uniform across all individuals, but instead differed for those in the lowest and highest cognitive categories (CCs). We identified that the magnitude of impact varied across cognitive category. The highest magnitudes of impact were observed in tasks that require greater levels of concentration, such as using computer and sewing or knitting. The factors in the central cognitive category appear to present a reduced magnitude of effects when compared to the impact on the remaining categories. Verification analyses confirmed that this pattern of results held (see Supplementary material for further details). These findings confirm our central hypothesis that lifestyle factors have different effects depending on one’s current level of memory function. With that said, we must interpret this effect cautiously as we cannot rule out the effects of reverse causation from the current data. For example, because individuals present higher cognitive categories they become more likely to take part in enriching lifestyles whereas others who are in poorer cognitive categories become more likely to take part in detrimental ones. Further studies using clinical trials should be conducted to investigate this question. Additional analysis should be performed to understand whether the impact of the factors on the central CC is reduced or if it they’re negligible. Should the former option be true, individuals in this category might require a greater investment to achieve similar degrees of memory change that individuals in other cognitive categories achieve more easily. Should the latter option be true, it is crucial to investigate why these individuals might be resistant to their environmental context. The possibility of this individual predisposition leads us to our second critical result–the analysis revealed dichotomous findings on individuals’ overall susceptibility to lifestyle factors. Individuals in the intermediate CC were largely resistant to the effects of lifestyle factors, be they detrimental or enriching. By comparison, individuals in the extreme CCs were especially susceptible to these same lifestyle factors.

An explanation for this pattern of results may come not from gerontology but from the developmental sciences. Boyce and Ellis (2005) advanced a theory that accounts for biological sensitivities in childhood to various harmful and protective environmental effects and their impact on development into adulthood. They proposed a developmental dichotomy to describe their pediatric patients: the theory of *orchids* and *dandelions*. According to this view, orchid individuals are more environment-sensitive: they thrive under ideal conditions but are also more susceptible to deterioration in poor environmental conditions [see Boyce and Ellis (2005) and Ellis et al. (2011)]. In contrast, dandelion individuals are relatively less environment-sensitive: they do not thrive to the same degree as orchid individuals in ideal conditions but are also more resilient to deterioration in poor environmental conditions (Luthar et al., 1993; Masten, 2001). Although concepts of orchid and dandelion individuals were first developed to account for different trajectories in childhood development, the present results suggest that a similar framework may also apply at the other end of the life continuum, with more- and less-environment-sensitive older adults. The extreme cognitive categories may reflect the environment-sensitive qualities of orchid older adults. Conversely, the stability of the central cognitive score category may represent the environment-insensitive qualities of dandelion older adults.

Future research could be proposed to investigate the limits and extend the potential application of these findings. First, it stands to reason that converging evidence should come from new, untested life factors, and possibly on other outcome measures beyond cognitive function. For example, similar patterns of results may apply to resiliency and mental health in aging. This is of particular relevance given the large number of studies focused on understanding the relationship between exposure to adverse life events and the development of psychopathology, and further why some individuals, in similar contexts, succumb to debilitating psychiatric disease whereas others age normally (Faye et al., 2018; Feder et al., 2019). This research could also be of interest in the study of drivers of depression and loneliness often reported by older adults (Cotten et al., 2014). Within this framework, further research is necessary to understand how age and cohort effects may play important mediating roles.

In addition, future research can explore the relationship between environment-sensitive and environment-resistant individuals in the context of personality traits. This important area of research would allow for an understanding of individual fundamental abilities, crucial for the adaptation to specific environmental conditions but also societal contexts. Similar work has been conducted on child and adolescent populations, highlighting that temperament traits and specific gene variants may be associated with heightened sensitivity to the environment (Lionetti et al., 2018; Pluess et al., 2018). How these findings translate to the aging population has not yet been explored. Beyond this, further studies are needed to assess the potential bridges between the neurodevelopmental and aging fields.

One potential limitation of our work is the use of variables that have been collected by the same individuals, 4 years apart. Given our research question, we identified the leave-behind questionnaire as an important section to include given the richness of lifestyle factors that had been collected, however, the attrition rates associated to this section are relatively high. The substantial reduction in observations could result in biasing our sample of data (D’Agostino, 1998). For example, excluded observations may be associated to individuals belonging to under-represented categories, therefore possibly reducing the generalizability of our results. However, when considered alternatives, such as imputation techniques, we found that these are suitable approaches when the number of observations that need replacement are relatively small (Engels and Diehr, 2003). In this study, given the dimension of missing data we found that imputation could introduce further biases and large measurement error-type problems (Engels and Diehr, 2003). To understand the risk of biases in our data, we conducted several analyses testing the representativeness of our sample. The results of those analysis suggested that our data is an acceptable representation of the population (Supplementary material: p. 4).

Taken on the whole, our findings offer a new conceptualization of the aging process: the *orchid* and *dandelion* aging theory. This new framework will allow researchers to formulate new questions and hypotheses and reinterpret the literature’s critical findings. These discoveries also offer new possibilities to help and support our older populations throughout the aging process. Understanding this distribution of the aging population could help decision-makers offer older adults solutions fitting their needs instead of the current one-size-fits-all policy model. The possibility of having a significant impact on aging health policies and providing substantial evidence for new social prescribing programs is real.

## Data availability statement

The original contributions presented in this study are included in the article/Supplementary material, further inquiries can be directed to the corresponding author.

## Author contributions

ER: full access to all of the data in the study, take responsibility for the integrity of the data and the accuracy of the data analysis, and statistical analysis. SM: obtain the funding. FF and SM: study supervision. All authors: study concept and design, acquisition, analysis, or interpretation of data, drafting of the manuscript, critical revision of the manuscript for important intellectual content, provided input into the design, edited and revised the manuscript, and read and approved the final manuscript.

## Footnotes

1 The code used for this analysis is available from authors upon request.
