# Supplementary material for: Does cognitive aging follow an orchid and dandelion phenomenon?
Source: Front Aging Neurosci. 2022 Oct 10;14:986262. doi: 10.3389/fnagi.2022.986262 (PMC9588970; doi:10.3389/fnagi.2022.986262)
Supplement: Supplementary file 1 [file Data_Sheet_1.docx]

**Does cognitive aging follow an orchid and dandelion phenomenon?**

Emma A. Rodrigues^a^, Gregory J. Christie^b^, Faranak Farzan^c^, Sylvain Moreno^a, b *^

^a^ School of Interactive Arts and Technology, Simon Fraser University, Surrey, BC, Canada

^b^ Digital Health Circle, Simon Fraser University, Surrey, BC, Canada

^c^ School of Mechatronics and Systems Engineering, Simon Fraser University, Surrey, BC, Canada

*Correspondence to Dr. Sylvain Moreno:

Email: [sylvain_moreno@sfu.ca](mailto:sylvain_moreno@sfu.ca)

Telephone: 778-782-9742

**Supplementary Material**

**Materials and Methods**

Software and Database

The software used was Stata 14.2.

The Health and Retirement Study (HRS)(*1*) (see website: https://hrs.isr.umich.edu/) is a longitudinal database that collects employment history, work history, disability, retirement plans, and net worth, income and health of U.S. population over the age of 50. Our goal was to use the HRS to evaluate variables preserving cognitive health in aging. We extracted cognitive health information along with life factors data from the HRS, as reported in the modules Preload, Physical Health, Leave-behind questionnaires, and Cognition of 2012 and 2016 waves. The dependent variable, cognitive health, was computed as the sum of the total number of words correctly remembered by the respondents during the immediate and delayed recalls in each of the waves.

Data description

The HRS database presents different question formats depending on the type of query. Specifically, the factors are collected in two main ways - in a binary format or in a Likert scale format with different answers and number of options. “How often do you do a vigorous activity?”, “How often do you read?”, and “Do you have an ongoing financial strain?” are examples of questions with different answering formats. Therefore, for simplification purposes, all variables were transformed into binary values, with the exception of age and education. Details on the binarization process can be found below.

Participants and selection criteria

As part of our selection criteria, we included individuals above the age of 60. In total, our representative dataset contained information on 20500 respondents characterized by 36 independent variables. We excluded respondents who had missing data for any of these previously listed factors^[[1]](#footnote-1)^. Our final number of respondents was 3530. The data was strongly balanced with 1752 male participants and 1778 female participants.

Data Analysis

Factor Selection

The selected lifestyle factors were defined based on existing literature (for references, see Supplementary Table 1). Supplementary Table 1 identifies key results in the literature that support the inclusion of these specific factors as relevant to cognitive health. Following this review of the literature, we decided to include 36 lifestyle factors.

The factors that display stronger statistical significance included: *Often read*, *Often do word games, Often sew/knit*, *Often use computer,* *Often do mild activities;* *Years of Education*, *Drinking*, *Often does hobby,* *Often walk 20 min, Age, Ongoing housing problems,* and *Smoking.* The following factors do not display any impact (i.e., were not statistically significant in our model) on the measure of cognitive health*: Ongoing health problems*, *Ongoing physical/emotional problem in spouse/child*, *Ongoing drug/alcohol problem with a family member*, *Ongoing difficulties at work*, *Ongoing financial strain*, *Ongoing problems in close relationship*, *Often do activities with grandchildren,* *Often volunteer with youth, Often do charity work, Often do education courses, Often attend non-religious organizations, Often pray privately, Often watch television, Often play cards and games, Often do writing, Often do maintenance/gardening, Often bake/cook, Often do Vigorous Activities* and *Often do Moderate Activities*.

Similar to the covariate selection process, the dependent variable was also identified based on prior literature and findings. With memory being one of the cognitive functions that most declines with age, we found it a sensitive measure to change over time (*2*). Then we selected a memory test that did not suffer from either ceiling or flooring effects to take advantage of the intrinsic data variability(*3*). To that end, we selected composite word recall (immediate + delayed) as a dependent variable. This is an ordered variable given that it was recorded based on the number of recalled words out of a list of ten. The results vary from zero to ten, with a greater concentration of answers around the centre of the scale.

Binarization

As priorly mentioned, different questions have different data collection processes. The binary format questions remained unchanged. The Likert format questions are represented here with three examples:

1. How often do you do physical activity?

Never; Hardly Ever; Sometimes; Often; Every day; Blank/not applicable;

1. How often do you do walk for 20 minutes or more? (as seen in Figure 1)

Daily; Several times a week; Once a week; Several times a month; At least once a month; Not in the last month; Never/Not relevant; Blank/not applicable;

1. Do you have an ongoing financial strain?

No, didn’t happen; yes, but not upsetting; yes, somewhat upsetting; yes, very upsetting; Blank/not applicable

The responses were merged to fit the criteria of 0, 1 or missing value if blank. The answers of Often or Everyday were merged and re-labeled as 1 and answers of Sometimes, Hardly Ever and Never were merged and re-labeled as 0. The answers of Daily or Several times a week were merged and re-labeled as 1 and answers of Once a week, Several times a month, At least once a month, Not in the last month and Never/Not relevant were merged and re-labeled as 0. Blank answers remained missing values. For the remainder of questions, No was re-labeled as 0 and Yes as 1 regardless of the degree of distress.

These first questions are about the activities in your life now. Please tell us

HOW OFTEN YOU DO EACH ACTIVITY. (Mark (X) one box for each line.)

Walk for 20 minutes or more?

.................................................................................

1411 1. DAILY

1469 2. SEVERAL TIMES A WEEK

519 3. ONCE A WEEK

665 4. SEVERAL TIMES A MONTH

444 5. AT LEAST ONCE A MONTH

881 6. NOT IN THE LAST MONTH

861 7. NEVER/NOT RELEVANT

14668 Blank. INAP (Inapplicable); Partial Interview; Missing

Figure 1 – Example of setting of questioning for HRS participants

Stratification

The original ordinality of the dependent variable was reflected in the ten categories representing the number of recalled words. Given the small number of observations in each of the original categories, we decided to merge them into groups of 20%. The bottom 20% was category 1, the second 20%, category 2, and so forth with the highest 20% being category 5. The sectioning process allowed us to define which data belonged to which category without the bias of the researcher. Furthermore, it avoided skewed results due to heterogeneous population concentrations.

Model Selection and Description

To address the research question, we used an ordinal logistic regression (OLR) model. As explained next, this model provides the most informative comparisons for the subject matter as well as the desired amount of model flexibility (*4*). In addition, due to the logistic nature of the distribution function, the exponential form of the regression coefficients can be interpreted as odds ratios (ORs) (*5*). The OLR is indicated when an originally continuous response variable is later grouped. This model allows us to compute the odds of an individual being at or below a category (*6*). The framework of the ordinal regression model is described in the following section.

Ordinal Logistic Regression Model

Given our ordinal response variable $y$, which represents the cognitive function, the categories can be ordered from a lower degree of cognition to a higher degree of cognition. $y_{i}$ will take a discrete value between 1 and 5 corresponding to the five categories in our study. For the purpose of estimation of the ordered logit model, we consider the latent variable representation on Equation (1), where $X_{i}$ is a $k\times1$ vector of covariates, such as those described in Supplementary Table 1, and $u$ is logistically distributed with Equation (2). Regarding the thresholds for classification of the model outcome by the respective categories, we define Equation (3). Moreover, the conditional probability of an individual being in a given category is computed as Equation (4).

The OLR is the most commonly used logistic regression model for an ordinal response, given that the effect of a covariate on $y$ can be quantified by one regression coefficient. Furthermore, it allows for the calculation of odds ratios in a clear and reliable manner which will assess the odds of an individual being in a given category instead of another. See, for instance, McCullagh (1980) for more details on the model estimation(*7*). Results on the OLR analysis can be found in the Supplementary Table 2 and 3 which present the overall estimation results.

**Notes**

Verifications Analysis

To investigate the robustness of the results obtained, we further analyzed different possibilities that may influence the results. Specifically, three main variants were considered.

- The impact of the number of participants per category

To address whether the number of participants in each cognitive category influenced the pattern of impacts provided by the analysis of the marginal effects, we selected the group with the smallest number of participants and randomly selected an equal number of participants in the remaining groups. This led to 560 participants in each cognitive category.

In this analysis, as seen in Figure 3, we obtained 9 statistically significant variables. *Age, Smoking* and *Housing Problems* appear to have a positive relation with the lowest CCs, and a negative relation with the highest CCs. Additionally, *Drinking, Word Games, Use Computer, Sew/Knit, Mild Activities* and *Education* appear to have a negative relation with the lowest CCs and a positive relation with the highest CCs.

Furthermore, the patterns obtained revealed that the highest impacts were observed in the lowest and highest CCs, and the smallest impacts were observed in the cognitive category 3.


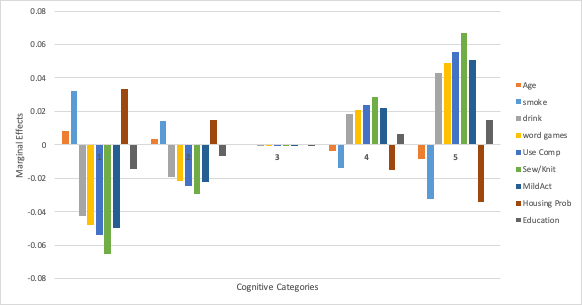


Equal Number of Participants per Category

Figure 3 - Marginal effects analysis with equal number of participants per category

- The impact of the stratification method

To address whether the number of categories created influenced the patterns of impacts, we re-ran the analysis with different stratification quantities. In specific, we compared the original stratification results (which included 5 cognitive categories) with results from new stratifications using 3, 4 and 9 cognitive categories.

- - 3 Cognitive Categories

In this analysis, as seen in Figure 4, we obtained 13 statistically significant variables. *Age, Attend Educational or Training courses, Smoking* and *Housing Problems* appear to have a positive relation with the lowest CCs, and a negative relation with the highest CCs. Additionally, *Drinking, Word Games, Use Computer, Sew/Knit, Mild Activities, Education, Do Maintenance, Attend Sports/Socials* and *Often Ail Friends/Family* appear to have a negative relation with the lowest CCs and a positive relation with the highest CCs.

The cognitive category in which the impact of the variables was smallest was 2. The cognitive category in which the impact of the variables was greatest was 1 and 3.


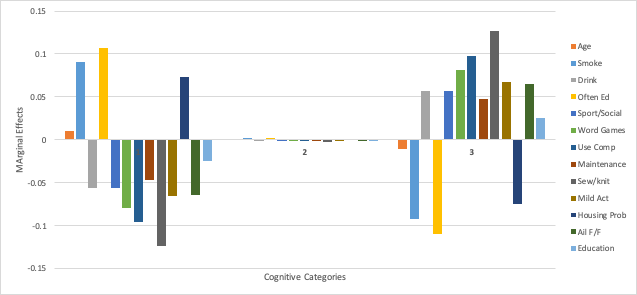


3 Cognitive Categories

Figure 4 - Marginal effects analysis with 3 cognitive categories

- - 4 Cognitive Categories

In this analysis, as seen in Figure 5, we obtained 10 statistically significant variables. *Age, Smoking, Walk 20 min* and *Housing Problems* appear to have a positive relation with the lowest CCs, and a negative relation with the highest CCs. Additionally, *Drinking, Word Games, Use Computer, Sew/Knit, Mild Activities* and *Education* appear to have a negative relation with the lowest CCs and a positive relation with the highest CCs.

The cognitive category in which the impact of the variables is smallest is 2, followed closely by category 3. The cognitive category in which the impact of the variables was greatest was 1 and 4.


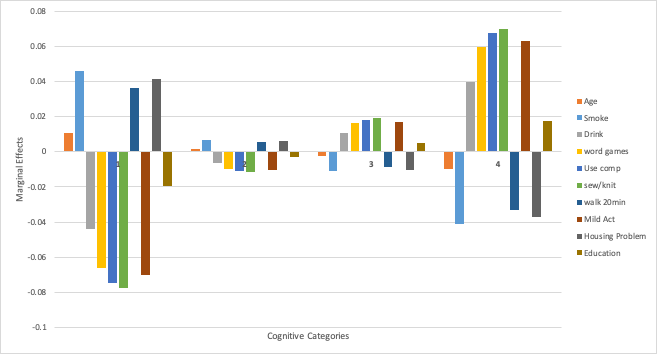


4 Cognitive Categories

Figure 5 - Marginal Effects analysis with 4 cognitive categories

- - 9 Cognitive Categories

In this analysis, as seen in Figure 6, we obtained 12 statistically significant variables. *Age, Smoking* and *Housing Problems* appear to have a positive relation with the lowest CCs, and a negative relation with the highest CCs. Additionally, *Drinking, Word Games, Use Computer, Sew/Knit, Mild Activities, Education, Play Card Games, Do Maintenance* and *Attend Sports/Socials* appear to have a negative relation with the lowest CCs and a positive relation with the highest CCs.

The cognitive category in which the impact of the variables is smallest is 5. The cognitive category in which the impact of the variables was greatest was 1 and 9.


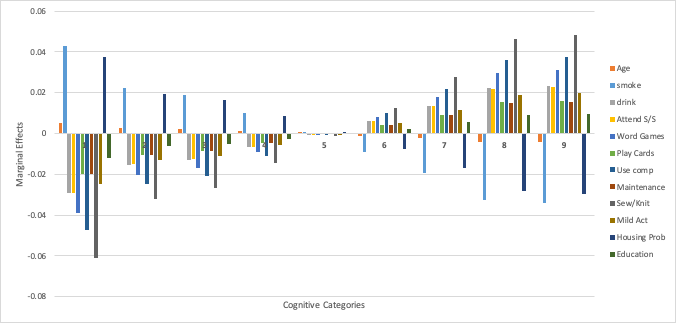


9 Cognitive Categories

Figure 6 - Marginal effects analysis with 9 cognitive categories

Note: Data can be made available upon request from authors.

**Table(s)**

Supplementary Table 1

List of covariates used in analysis

| **Covariate** | **Reference (e.g.)** | **Covariate** | **Reference (e.g.)** |
| --- | --- | --- | --- |
| Ongoing health problems | (*8*–*10*) | Often do word games | (*11*)^,(<i>12</i>)12^ |
| Ongoing physical/emotional problem in spouse/child | (*13*, *14*) | Often play cards and games | (*15*, *16*) |
| Ongoing drug/alcohol problem with family member | (*17*, *18*) | Often do writing | (*19*) |
| Ongoing difficulties at work | (*20*) | Often use computer | (*21*) |
| Ongoing financial strain | (*22*, *23*) | Often do maintenance/gardening | (*24*) |
| Ongoing housing problem | (*22*, *25*, *26*) | Often bake/cook | (*27*) |
| Ongoing problems in close relationship | (*28*) | Often sew/knit | (*27*, *29*) |
| Often do activities with grandchildren | (*30*, *31*) | Often walk for 20 min | (*32*) |
| Often volunteer with youth | (*33*) | Often do Vigorous Activities | (*34*, *35*) |
| Often do charity work | (*36*) | Often do Moderate Activities | (*34*, *35*) |
| Often do education courses | (*37*–*39*) | Often do Mild Activities | (*34*, *35*) |
| Often attend non-religious organizations | (*40*) | Age | (*41*, *42*) |
| Often pray privately | (*43*) | Years of Education | (*25*, *44*) |
| Often read | (*45*, *46*) | Smokes | (*47*, *48*) |
| Often watch television | (*46*, *49*) | Drinks | (*50*) |
| Often do hobby | (*51*) | Often attend sports/socials/clubs | (*52*) |
| Often care adult | (*53*) | Often play sports/exercise | (*54*) |
| Ongoing difficulty paying bills | (*55*) | Regularly help ailing family/friends | (*56*) |

Supplementary Table 1 -Description of 36 covariots included in analysis and supporting references

Supplementary Table 2

Descriptive results of OLR analysis

| $y$**: Cognitive Score** | **Coeff.** | **Std. Err.** | **z** | **P>\|z\|** | **[95% Conf. Interval]** | |
| --- | --- | --- | --- | --- | --- | --- |
| Vigorous Activities | .0692738 | .0796318 | 0.87 | 0.384 | -.0868016 | .2253493 |
| Moderate Activities | .0692161 | .0711224 | 0.97 | 0.330 | -.0701812 | .2086134 |
| Mild Activities | .3340872 | .0648227 | 5.15 | 0.000 | .2070371 | .4611374 |
| Often care adult | .0321184 | .1010543 | 0.32 | 0.751 | -.1659444 | .2301812 |
| Often do activities with grandchildren | -.0363207 | .0711624 | -0.51 | 0.610 | -.1757965 | .1031551 |
| Often volunteer with youth | .0495151 | .1463767 | 0.34 | 0.735 | -.237378 | .3364083 |
| Often do charity work | .1006993 | .1048294 | 0.96 | 0.337 | -.1047625 | .3061611 |
| Often attend educational or training courses | .1763935 | .180789 | 0.98 | 0.329 | -.1779465 | .5307334 |
| Often attend sports/social/club | .0798489 | .0897617 | 0.89 | 0.374 | -.0960808 | .2557786 |
| Often attend non-religious orgs | -.0088818 | .1524001 | -0.06 | 0.954 | -.3075804 | .2898169 |
| Often pray privately | .048743 | .0611128 | 0.80 | 0.425 | -.0710358 | .1685219 |
| Often read | .2447138 | .0804649 | 3.04 | 0.002 | .0870055 | .4024222 |
| Often watch television | .1078919 | .1815644 | 0.59 | 0.552 | -.2479678 | .4637516 |
| Often do word games | .3720905 | .0627438 | 5.93 | 0.000 | .2491149 | .4950661 |
| Often play cards and games | .0826794 | .0754452 | 1.10 | 0.273 | -.0651905 | .2305493 |
| Often do writing | .133298 | .0871046 | 1.53 | 0.126 | -.0374238 | .3040199 |
| Often use computer | .5144185 | .0687941 | 7.48 | 0.000 | .3795845 | .6492525 |
| Often do maintenance/gardening | .1031092 | .0636428 | 1.62 | 0.105 | -.0216284 | .2278469 |
| Often bake/cook | -.0008383 | .0633692 | -0.01 | 0.989 | -.1250397 | .1233631 |
| Often sew/knit | .4375562 | .1351585 | 3.24 | 0.001 | .1726504 | .702462 |
| Often do hobby | -.1473654 | .0726733 | -2.03 | 0.043 | -.2898025 | -.0049284 |
| Often play sports/exercise | -.0093236 | .0743148 | -0.13 | 0.900 | -.1549781 | .1363308 |
| Often walk for 20 min | -.1798729 | .0683998 | -2.63 | 0.009 | -.313934 | -.0458118 |
| Ongoing difficulty paying bills | -.0600553 | .0705799 | -0.85 | 0.395 | -.1983895 | .0782789 |
| Self ongoing health problems | -.0309276 | .0689672 | -0.45 | 0.654 | -.1661009 | .1042457 |
| Ongoing phy/emot problems in sp/chld | .0864745 | .0654955 | 1.32 | 0.187 | -.0418943 | .2148432 |
| Ongoing drug/alcohol probs fam mbr | -.0637864 | .0793697 | -0.80 | 0.422 | -.2193481 | .0917754 |
| Ongoing difficulties at work | -.0281635 | .0940062 | -0.30 | 0.764 | -.2124122 | .1560852 |
| Ongoing financial strain | .0315049 | .077851 | 0.40 | 0.686 | -.1210802 | .18409 |
| Ongoing housing problems | -.2997227 | .0871656 | -3.44 | 0.001 | -.4705641 | -.1288812 |
| Ongoing problems close relationship | -.0048672 | .0759421 | -0.06 | 0.949 | -.1537109 | .1439765 |
| Reg help ailing friend/fam | .0748511 | .0681212 | 1.10 | 0.272 | -.058664 | .2083663 |
| Drinking | .2683895 | .0625742 | 4.29 | 0.000 | .1457463 | .3910327 |
| Smoking | -.2603079 | .0807091 | -3.23 | 0.001 | -.4184949 | -.1021209 |
| Age | -.0671034 | .0042736 | -15.70 | 0.000 | -.0754795 | -.0587274 |
| Years of Education | .1323413 | .0121925 | 10.85 | 0.000 | .1084445 | .1562381 |

Supplementary Table 2 -Discriptive results of OLR analysis including β coefficients, standard errors and P values

Supplementary Table 3

Descriptive results of OLR analysis

| **Thresholds** |  |  |  |  |
| --- | --- | --- | --- | --- |
| $\alpha_{1}$ | -3.827573 | .398016 | -4.60767 | -3.047476 |
| $\alpha_{2}$ | -2.618773 | .3953839 | -3.393711 | -1.843834 |
| $\alpha_{3}$ | -1.414998 | .3939296 | -2.187086 | -.6429104 |
| $\alpha_{4}$ | -.0578471 | .3939877 | -.8300488 | .7143545 |

Supplementary Table 3 -Discriptive results of OLR analysis including β coefficients and standard errors for the calculated thresholds

**Equations**

${y_{i}}^{*}=\beta{X'}_{i}+ u_{i} , i=1,\ldots, N.$ (1)

$\Lambda\left( z \right)=\frac{e^{z}}{{1+e}^{z}}$ (2)

$$y_{i}=1 if {{y_{i}}^{*}\leq\alpha}_{1}$$

$$y_{i}=2 if {{{\alpha_{1}<y}_{i}}^{*}<\alpha}_{2}$$

$y_{i}=3 if {{{\alpha_{2}<y}_{i}}^{*}<\alpha}_{3}$ (3)

$$y_{i}=4 if {{{\alpha_{3}<y}_{i}}^{*}<\alpha}_{4}$$

$$y_{i}=5 if {{y_{i}}^{*}\geq\alpha}_{4}$$

$$\Pr\left[ y_{i}=1 | X \right]=\wedge\left( \alpha_{1}-\beta{X^{'}}_{i} \right)$$

$$\Pr\left[ y_{i}=2 | X \right]=\wedge\left( \alpha_{2}-\beta{X^{'}}_{i} \right)-\wedge(\alpha_{1}-\beta{X^{'}}_{i})$$

$\Pr\left[ y_{i}=3 | X \right]=\wedge\left( \alpha_{3}-\beta{X^{'}}_{i} \right)-\wedge(\alpha_{2}-\beta{X^{'}}_{i})$ (4)

$$\Pr\left[ y_{i}=4 | X \right]=\wedge\left( \alpha_{4}-\beta{X^{'}}_{i} \right)-\wedge(\alpha_{3}-\beta{X^{'}}_{i})$$

$$\Pr\left[ y_{i}=5 | X \right]=1-\wedge(\alpha_{4}-\beta{X^{'}}_{i})$$

Source: Greene, W.H., & Hensher, D. A. (2010). Modeling ordered choices a primer. Cambridge University Press. pp 83-85

**References**

1. Health and Retirement Study, (available at https://hrs.isr.umich.edu/about).

2. A. Sixsmith, J. Sixsmith, M. L. Fang, B. Horst, *AgeTech, Cognitive Health and Dementia* (Morgan & Claypool Publishers, ed. 1st, 2020).

3. L. B. Brown, M. Storandt, Sensitivity of category cued recall to very mild dementia of the Alzheimer type. *Arch. Clin. Neuropsychol.* **15**, 529–534 (2000).

4. M. W. Fagerland, adjcatlogit, ccrlogit, and ucrlogit: Fitting ordinal logistic regression models. *Stata J.* **14**, 947–964 (2014).

5. D. W. Hosmer, S. Lemeshow, R. X. Sturdivant, *Ordinal logistic regression models. Applied logistic regression* (John Wiley & Sons, ed. 3rd, 2013).

6. M. N. S. Abreu, A. L. Siqueira, C. S. Cardoso, W. T. Caiaffa, Ordinal logistic regression models: Application in quality of life studies. *Cad. Saude Publica*. **24** (2008), doi:10.1590/s0102-311x2008001600010.

7. P. McCullagh, Regression Models for Ordinal Data. *R. Stat. Soc.* **42**, 109–142 (1980).

8. M. Choi, M. C. Lohman, B. Mezuk, Trajectories of cognitive decline by driving mobility: Evidence from the Health and Retirement Study. *Int. J. Geriatr. Psychiatry*. **29**, 447–453 (2014).

9. T. W. Auyeung, J. S. W. Lee, T. Kwok, J. Woo, Physical frailty Predicts future cognitive decline – a four-year ProsPective study in 2737 cognitively normal older adults. *J. Nutr. Health Aging* (2011).

10. L. F. Schumpf, N. Theill, D. A. Scheiner, D. Fink, F. Riese, C. Betschart, Urinary incontinence and its association with functional physical and cognitive health among female nursing home residents in Switzerland. *BMC Geriatr.* **17**, 17 (2017).

11. E. M. Zelinski, R. Reyes, Cognitive benefits of computer games for older adults. *Gerontechnology*. **8**, 220–235 (2009).

12. E. M. Zelinski, Games and Other Training Interventions to Improve Cognition in Healthy Older Adults. *Transform. Gaming Comput. Simul. Technol. across Ind.*, 192–205 (2017).

13. K. B. Dassel, D. C. Carr, P. Vitaliano, Does Caring for a Spouse With Dementia Accelerate Cognitive Decline? Findings From the Health and Retirement Study. *Gerontologist*. **57**, 319–328 (2017).

14. M. M. Pertl, B. A. Lawlor, I. H. Robertson, C. Walsh, S. Brennan, Risk of Cognitive and Functional Impairment in Spouses of People With Dementia: Evidence From the Health and Retirement Study. *J. Geriatr. Psychiatry*. **28(4)**, 260–271 (2015).

15. M. Perquin, M. Vaillant, A.-M. Schuller, J. Pastore, J.-F. Dartigues, M.-L. Lair, N. Diederich, Lifelong Exposure to Multilingualism: New Evidence to Support Cognitive Reserve Hypothesis. *PLoS One*. **8**, e62030 (2013).

16. C. Y. Kuo, Y. M. Huang, Y. Y. Yeh, Let’s play cards: Multi-component cognitive training with social engagement enhances executive control in older adults. *Front. Psychol.* **9**, 1–13 (2018).

17. J. M. Campbell, T. P. Oei, A cognitive model for the intergenerational transference of alcohol use behavior. *Addict. Behav.* **35**, 73–83 (2010).

18. L. Lander, J. Howsare, M. Byrne, The Impact of Substance Use Disorders on Families and Children: From Theory to Practice. *Soc. Work Public Health*. **23**, 1–7 (2013).

19. R. Wilson, P. Boyle, L. Yu, L. Barnes, J. Schneider, D. Bennett, Life-span cognitive activity, neuropathologic burden and cognitive aging. *Am. Acad. Neurol.* **9**, P625–P625 (2013).

20. D. Van Der Linden, G. P. J. Keijsers, P. Eling, R. Van Schaijk, Work stress and attentional difficulties: An initial study on burnout and cognitive failures. *Work Stress*. **19**, 23–36 (2005).

21. D. Calhoun, S. B. Lee, Computer usage and cognitive capability of older adults: Analysis of data from the Health and Retirement Study. *Educ. Gerontol.* **45**, 22–33 (2019).

22. J. J. McArdle, Longitudinal Dynamic Analyses of Cognition in the Health and Retirement Study Panel. *Adv. Stat. Anal.* **95**, 453–480 (2011).

23. R. W. Schroeder, P. K. Martin, A. Walling, Neuropsychological Evaluations in Adults. *Am. Acad. Fam. Physicians* (2019) (available at https://web.a.ebscohost.com/ehost/pdfviewer/pdfviewer?vid=1&sid=9d117707-7137-455f-8c6b-e4cebb2f9f6e%40sdc-v-sessmgr02).

24. S. A. Park, A. Y. Lee, H. G. Park, W. L. Lee, Benefits of gardening activities for cognitive function according to measurement of brain nerve growth factor levels. *Int. J. Environ. Res. Public Health*. **16** (2019), doi:10.3390/ijerph16050760.

25. Y. Stern, B. Gurland, T. K. Tatemichi, M. X. Tang, D. Wilder, R. Mayeux, Influence of Education and Occupation on the Incidence of Alzheimer’s Disease. *JAMA J. Am. Med. Assoc.* **271**, 1004 (1994).

26. A. Z. Burzynska, L. H. Malinin, Enriched Environments for Healthy Aging: Qualities of Seniors Housing Designs Promoting Brain and Cognitive Health. *Seniors Hous. Care J.* **25**, 15–37 (2017).

27. A. Iizuka, H. Suzuki, S. Ogawa, K. E. Kobayashi-Cuya, M. Kobayashi, T. Takebayashi, Y. Fujiwara, Can cognitive leisure activity prevent cognitive decline in older adults? A systematic review of intervention studies. *Geriatr. Gerontol. Int.* **19**, 469–482 (2019).

28. B. Laursen, W. M. Bukowski, A Developmental Guide to the Organisation of Close Relationships. *Int. J. Behav. Dev.* **21**, 747–770 (1997).

29. J. Riley, B. Corkhill, C. Morris, The benefits of knitting for personal and social wellbeing in adulthood: Findings from an international survey. *Br. J. Occup. Ther.* **76**, 50–57 (2012).

30. K. F. Burn, V. W. Henderson, D. Ames, L. Dennerstein, C. Szoeke, Role of grandparenting in postmenopausal women’s cognitive health. *Menopause*. **21**, 1069–1074 (2014).

31. K. Burn, C. Szoeke, Grandparenting predicts late-life cognition: Results from the Women’s Healthy Ageing Project. *Mauritas*. **82**, 317–322 (2015).

32. H. Makizako, K. Tsutsumimoto, T. Doi, R. Hotta, S. Nakakubo, T. Liu-Ambrose, H. Shimada, Effects of exercise and horticultural intervention on the brain and mental health in older adults with depressive symptoms and memory problems: study protocol for a randomized controlled trial [UMIN000018547]. *Trials*. **16**, 499 (2015).

33. J. W. K. Yeung, Z. Zhang, T. Y. Kim, Volunteering and health benefits in general adults: Cumulative effects and forms. *BMC Public Health*. **18**, 1–8 (2017).

34. S. Sabia, A. Dugravot, J. F. Dartigues, J. Abell, A. Elbaz, M. Kivimäki, A. Singh-Manoux, Physical activity, cognitive decline, and risk of dementia: 28 year follow-up of Whitehall II cohort study. *BMJ*. **357**, 1–10 (2017).

35. Y. T. Chang, Physical Activity and Cognitive Function in Mild Cognitive Impairment. *ASN Neuro*. **12** (2020), doi:10.1177/1759091419901182.

36. F. J. Infurna, M. A. Okun, K. J. Grimm, Volunteering Is Associated with Lower Risk of Cognitive Impairment. *J. Am. Geriatr. Soc.* **64**, 2263–2269 (2016).

37. R. Y. Wood, K. K. Giuliano, C. U. Bignell, W. W. Pritham, Assessing Cognitive Ability in Research: Use of MMSE with Minority Populations and Elderly Adults with Low Education Levels. *J. Gerontol. Nurs.* **32**, 45–54 (2006).

38. X. Meng, C. D’Arcy, Education and dementia in the context of the cognitive reserve hypothesis: A systematic review with meta-analyses and qualitative analyses. *PLoS One*. **7** (2012), doi:10.1371/journal.pone.0038268.

39. B. N. Guerra-Carrillo, K. Katovich, S. A. Bunge, Does higher education hone cognitive functioning and learning efficacy? Findings from a large and diverse sample. *PLoS One* (2017), doi:10.1371/journal.pone.0182276.

40. M. E. Kelly, H. Duff, S. Kelly, J. E. McHugh Power, S. Brennan, B. A. Lawlor, D. G. Loughrey, The impact of social activities, social networks, social support and social relationships on the cognitive functioning of healthy older adults: A systematic review. *Syst. Rev.* **6** (2017), doi:10.1186/s13643-017-0632-2.

41. A. Perry, W. Wen, N. A. Kochan, A. Thalamuthu, P. S. Sachdev, M. Breakspear, The independent influences of age and education on functional brain networks and cognition in healthy older adults. *Hum. Brain Mapp.* **38**, 5094–5114 (2017).

42. M. Zhang, R. Katzman, D. Salmon, H. Jin, G. Cai, Z. Wang, G. Qu, I. Grant, E. Yu, P. Levy, M. R. Klauber, W. T. Liu, The prevalence of dementia and Alzheimer’s disease in Shanghai, China: Impact of age, gender, and education. *Ann. Neurol.* **27**, 428–437 (1990).

43. S. Hosseini, A. Chaurasia, M. Cooke, M. Oremus, Effect of religious involvement on cognition from a life-course perspective: Protocol for a systematic review and meta-analysis. *BMJ Open*. **6**, 1–4 (2016).

44. P. Satz, H. Morgenstern, E. N. Miller, O. A. Selnes, J. C. McArthur, B. A. Cohen, J. Wesch, J. T. Becker, L. Jacobson, L. F. D’Elia, Low education as a possible risk factor for cognitive abnormalities in HIV-1: findings from the multicenter AIDS Cohort Study (MACS). *J. Acquir. Immune Defic. Syndr.* **6** (1993), pp. 503–11.

45. S. L. Connelly, L. Hasher, R. T. Zacks, Age and reading: The impact of distraction. *Psychol. Aging*. **6**, 533–541 (1991).

46. C. Berezuk, K. K. Zakzanis, J. Ramirez, A. C. Ruocco, J. D. Edwards, B. L. Callahan, S. E. Black, Functional Reserve: Experience Participating in Instrumental Activities of Daily Living is Associated with Gender and Functional Independence in Mild Cognitive Impairment. *J. Alzheimer’s Dis.* **58**, 425–434 (2017).

47. S. Knecht, H. Wersching, H. Lohmann, K. Berger, E. B. Ringelstein, How much does hypertension affect cognition?: Explained variance in cross-sectional analysis of non-demented community-dwelling individuals in the SEARCH study. *J. Neurol. Sci.* **283**, 149–152 (2009).

48. H. M. Fillit, R. N. Butler, A. W. O’Connell, M. S. Albert, J. E. Birren, C. W. Cotman, W. T. Greenough, P. E. Gold, A. F. Kramer, L. H. Kuller, T. T. Perls, B. G. Sahagan, T. Tully, Achieving and Maintaining Cognitive Vitality With Aging. *Mayo Clin. Proc.* **77**, 681–696 (2002).

49. C. Owsley, G. J. McGwin, M. E. Sloane, B. T. Stalvey, J. Wells, Timed instrumental activities of daily living tasks: Relationship to visual function in older adults. *Optom. Vis. Sci.* **78**, 350–359 (2001).

50. a Pfefferbaum, E. V Sullivan, M. J. Rosenbloom, D. H. Mathalon, K. O. Lim, A controlled study of cortical gray matter and ventricular changes in alcoholic men over a 5-year interval. *Arch. Gen. Psychiatry*. **55**, 905–12 (1998).

51. T. F. Hughes, R. Andel, B. J. Small, A. R. Borenstein, J. A. Mortimer, The Association Between Social Resources and Cognitive Change in Older Adults: Evidence From the Charlotte County Healthy Aging Study. *J. Gerontol. Psychol. Sci.* **63B**, 241–244 (2008).

52. J. Hwang, S. Park, S. Kim, Effects of participation in social activities on cognitive function among middle-aged and older adults in Korea. *Int. J. Environ. Res. Public Health*. **15** (2018), doi:10.3390/ijerph15102315.

53. M. G. Austrom, Y. Lu, Long Term Caregiving: Helping Families of Persons with Mild Cognitive Impairment Cope. *Curr. Alzheimer Res.* **23**, 392–398 (2009).

54. A. L. Busse, G. Gil, J. M. Santarém, W. J. Filho, Physical activity and cognition in the elderly: A review. *Dement. e Neuropsychol.* **3**, 204–208 (2009).

55. A. Mani, S. Mullainathan, E. Shafir, J. Zhao, Poverty impedes cognitive function. *Science (80-. ).* **342**, 976–981 (2013).

56. P. A. Thomas, H. Liu, D. Umberson, Family Relationships and Well-Being. *Innov. Aging*. **1**, 1–11 (2017).

1. “We acknowledge that the substantial reduction in observations may contribute to biasing the sample. We understand that the excluded observations may be associated to individuals belonging to under-represented categories, therefore possibly reducing the generalizability of our results. However, when considered alternatives, such as imputation techniques, we found that these are suitable approaches when the number of observations that need replacement are relatively small. In this study, given the dimension of missing data we found that imputation could introduce further biases and large measurement error-type problems. We also tested the representativeness of our sample by conducting several verifications analysis (supplementary material: p. 4).” [↑](#footnote-ref-1)
